# Development of a comprehensive and clinically applicable novel projection classification system for anterior communicating artery aneurysms

**DOI:** 10.1007/s10143-023-02275-y

**Published:** 2024-01-10

**Authors:** Metin Orakdogen, Orkhan Mammadkhanli, Baris Chousein, Osman Simsek

**Affiliations:** 1 Department of Neurosurgery, Trakya University School of Medicine, Trakya University Hospital, 22030 Balkan Campus, Edirne, Turkey; 2 Department of Neurosurgery, Private Kesan Hospital, Büyük Cami Neighborhood, 22800 Keşan, Edirne Turkey

**Keywords:** Acom aneurysm surgery, Projection classification, Linear, Quadrant

## Abstract

Various surgical and anatomical classifications have been proposed to date related to ACoA aneurysm projection. Nonetheless, a universally accepted classification system is yet to be established. This study is aimed at establishing a standardized classification system for ACoA aneurysms with utilization 3D technology and defining reference lines for their projections. The goal is to create a simple, understandable, surgically beneficial, and reliable classification system based on neurovascular structures in the region, including safe and hazardous zones. The radiologic data of 96 patients with ACoA aneurysm who were treated in our university hospital between 2012 and 2020 were retrospectively analyzed, and a planned classification scale was developed with the data obtained. The classification aimed to create 9 main projection groups in the sagittal plane: superior, inferior, anterior, and posterior in linear orientation, and anterosuperior, posterosuperior, anteroinferior, posteroinferior, and complex in quadrant orientation. The coronal and axial planes included medial, lateral, and midline classifications, resulting in a 3-dimensional classification system with 25 projections. Among the 96 patients, 32 had linear and 64 had quadrant projections. In the sagittal plane, the linear projection breakdown was as follows: superior (28%), inferior (6.25%), anterior (53%), and posterior (12.5%). For the quadrant projection, the distribution was as follows: anterosuperior (53%), posterosuperior (12.5%), anteroinferior (21.87%), posteroinferior (3.12%), and complex (9.37%). Overall, 35.4% aneurysms were anterosuperior, 17.7% anterior, 14.58% anteroinferior, 9.37% superior, 8.3% posterosuperior, 6.25% complex, 4.16% posterior, 2.08% posteroinferior, and 2.08% inferior projection. Our study proposes a projection classification that utilizes 3D technology for safe surgery based on neurovascular structures in the region and thus better reveals safe and hazardous zones, including three plans, three dimensions, and two orientations. The use of this classification system offers valuable guidance for daily practice in the treatment of ACoA aneurysms.

## Introduction

The projection of anterior communicating artery (ACoA) aneurysms plays a crucial role in determining surgical strategies, complexity, precautions, complications, and prognosis. Therefore, preoperative knowledge of ACoA projection is essential [1–16].

Numerous surgical and anatomical classifications based on cerebral angiography imaging have been proposed for ACoA aneurysm projection, particularly in the sagittal plane. However, a universally accepted classification system is yet to be established, and the criteria for reference line determination vary among these classifications [1–3, 5, 6, 8–14, 17–35] (Table 1).

**Table 1:** ACoA projection classification systems

Two dimensional (2D)				
Orientation	Surgical	Anatomical		
Base	Linear	Linear		Quadrant
Sagittal	Yaşargil (1984)^21^ Solomon (2001)^24^ Lukui Chen (2009)^18^ Wang (2015)^25^	Proust (2003)^12^ Birknes (2006)^30^ Hernesniemi (2008)^19^ Agrawal (2008)^1^ Gonzalez (2008)^35^ Hyun (2010)^20^ Huang (2011)^36^	Lawton (2011)^11^ Petraglia (2011)^22^ Cai (2015)^26^ Shao (2016)^29^ Kato (2019)^10^ Bohnstedt (2019)^2^ Bhattarai (2020)^17^	Diraz (1993)^5^ Suzuki (2008)^14^ Ivan (2019)^8^ Chen (2020)^3^ Junhui Chen, 2020^3^
Axial		Nathal (1992)^42^		
Unclassified	Riina (2002)^23^ Sekhar (2007)^13^ Moon (2015)^43^			
Three dimensional (3D)				
Orientation	Anatomical			
Base	Linear	Quadrant	Linear + quadrant	Octan
Sagittal	Matsukawa (2013)^28^ Kasinathan (2019)^9^	Nossek (2016) ^31^	Samson & Batjer (2012)^32^ Orakdöğen* (2023)	Ivan (2019)^8^
Coronal	Matsukawa (2013)^28^ Choi (2016) ^27^ Kasinathan (2019)^9^		Samson & Batjer (2012)^32^ Orakdöğen* (2023)	Ivan (2019)^8^
Axial	Choi (2016)^27^	Inagawa (1999)^6^	Orakdöğen* (2023)	

* Present study

With the advancements in 3D imaging techniques such as computed tomography angiography (CTA), magnetic resonance angiography (MRA), and digital subtraction angiography (DSA), a comprehensive assessment of cerebrovascular structures and aneurysm projection can now be achieved in a global 360-degree perspective. However, none of the existing classifications consider neurovascular structures as reference lines, nor do they evaluate the three planes (sagittal, coronal, and axial) and two orientations (linear and quadrant) in 3D. Thus, it is valuable to review current classifications and develop a unified system that encompasses these assessments.

In this study, we aimed to develop a novel classification system for ACoA aneurysms that utilizes 3D technology to establish reference lines based on neurovascular structures, effectively delineating safe and hazardous zones, thereby addressing a current gap and providing valuable insights for surgeons while enhancing the safety and effectiveness of surgical interventions.

## Materials and methods

After approval of local ethical committee (number: TÜTF-GOBAEK 2023/256), we retrospectively analyzed the radiological data of 96 ACoA aneurysm patients with single or multiple aneurysms, ruptured/unruptured, diagnosed with at least one of 3D CTA, MRA, and DSA examinations at Trakya University Faculty of Medicine, Department of Neurosurgery, between 2012 and 2020. Those with conventional 2D DSA examinations were excluded from the study.

### Definition of classification

**Fig. 1:**
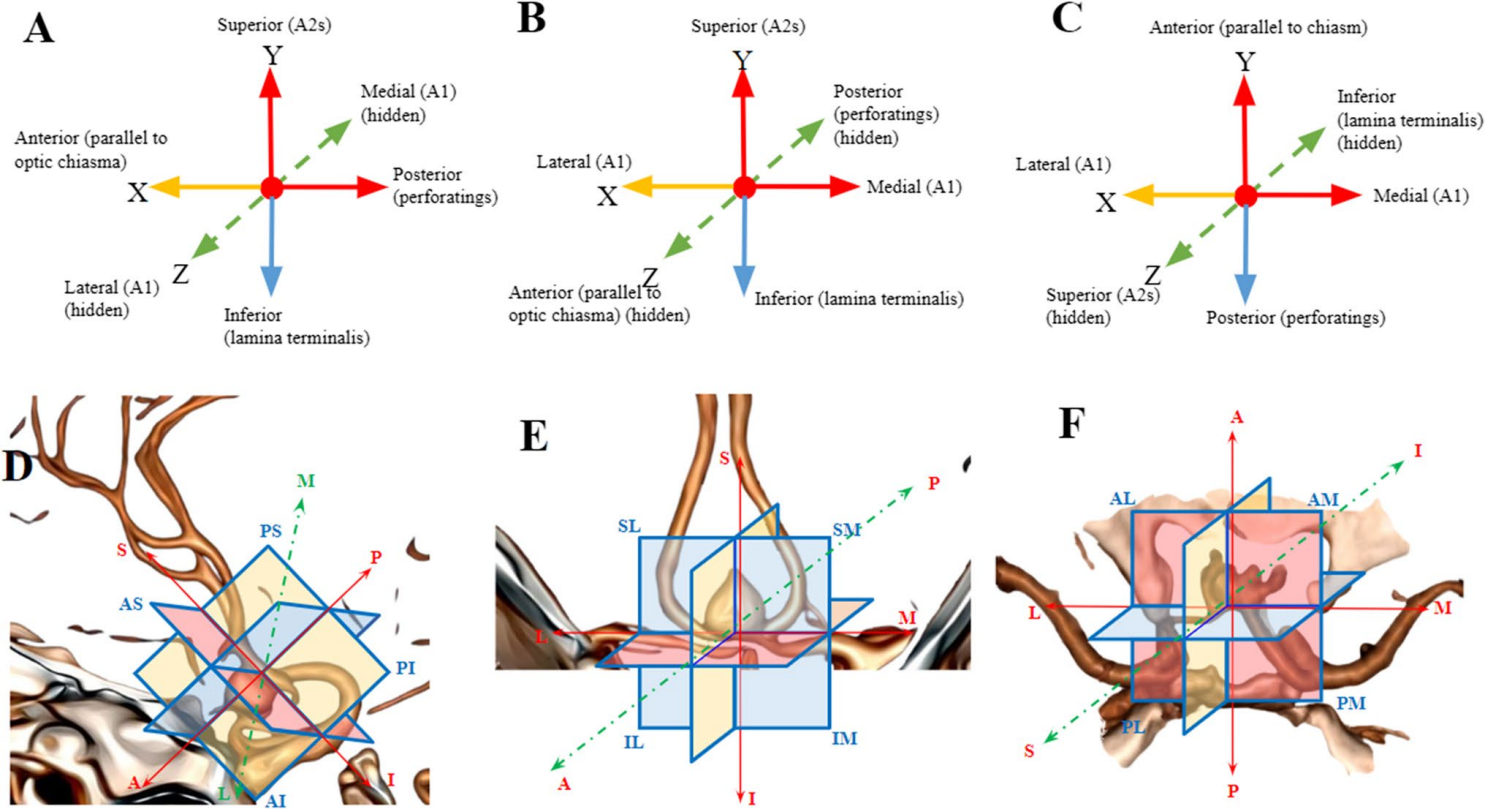
**A, D** Reference lines and 3D projections based on neurovascular structures in the sagittal plane. **B, E** Reference lines and 3D projections based on neurovascular structures in the coronal plane. **C, F** Reference lines and 3D projections based on neurovascular structures in the axial plane. Abbreviations: S, superior; M, medial; I, inferior; P, posterior; L, lateral; An, anterior; AS, anterosuperior; AI, anteroinferior; IL, inferolateral; IM, inferomedial; PI, posteroinferior; SM, superomedial; AM, anteromedial; AL, anterolateral

In determining the reference lines used in the classification, the most important neurovascular structures in the region that affect the clipping strategy of ACoA aneurysm, which need to be visualized and protected, are A1 and A2 segments, perforating arteries, optic chiasm, and lamina terminalis. These structures were taken as the basis (Fig. 1A–C).

The 3D aneurysm projection classification was performed according to these reference lines in three planes: sagittal, coronal, and axial in anatomical orientation and in two orientations, namely, linear and quadrant. Based on the coordinates and reference lines in Fig. 1, angiographic/schematic views of vascular structures in the ACoA complex in sagittal, coronal, and axial planes were created and aneurysm projections were determined.

The evaluation of ACoA aneurysm projections involved the use of anteroposterior (*X*), superoinferior (*Y*), and mediolateral (hidden) (*Z*) coordinates in the sagittal plane, mediolateral (*X*), superoinferior (*Y*), and anteroposterior (hidden) (*Z*) coordinates in the coronal plane, and mediolateral (*X*), anteroposterior (*Y*), and superoinferior (hidden) (*Z*) coordinates in the axial plane.

In the sagittal plane, the reference lines were based on a line parallel to the chiasm as the *X* coordinate and a line perpendicular to the A2s from the ACoA as the *Y* coordinate. The anterior projection evaluation considered the A2 segment in the closed A2 fork, ensuring that the aneurysm should be located in front of both A2s. The evaluation of the AP line in the inferior quadrant was based on the continuation of the reference line passing through the proximal A2 line in the superior quadrant, taking into account that the ACoA perforans exit at right angles to A2 and perpendicular to the lamina terminalis. In this plane, the *X* coordinate was used for superior-inferior aneurysms (superior above the *X* coordinate and inferior below the *X* coordinate), and the *Y* coordinate was used for anterior–posterior projection aneurysms (anterior aneurysms anterior to the *Y* coordinate and posterior aneurysms posterior to the *Y* coordinate). The *Z* coordinate, passing through the ACoA perpendicular to the *X* and *Y* coordinates and remaining hidden, was not utilized in this plane as it does not allow evaluation of medial, lateral, and midline projections.

In the sagittal plane, aneurysm projections on the reference lines were evaluated in linear orientation as superior, inferior, anterior, or posterior. Aneurysm projections between the reference lines were evaluated in quadrant orientation, resulting in anterosuperior, posterosuperior, anteroinferior, and posteroinferior quadrants.

In the coronal plane, the *X* coordinate was determined by the distal A1/Heubner-proximal A2 junction, ACoA, and the line passing perpendicular to the proximal A2s and parallel to the ACoA perforans known to be parallel to the ACoA. The *Y* coordinate was based on the line passing through the ACoA in the midline and parallel to the A2s and the lamina terminalis. In this plane, the *X* coordinate was used to categorize superior-inferior aneurysms (superior above the *X* coordinate and inferior below the *X* coordinate), and the *Y* coordinate was used for aneurysms with median, lateral, and midline projections (midline above the *Y* coordinate, lateral above the *Y* coordinate, and medial according to A1 dominance and approach side). The *Z* coordinate, which passes through the ACoA perpendicular to the *X* and *Y* coordinates and remains hidden, was only used for the evaluation of anterior projections. Aneurysms located in front of the ACoA were categorized as anterior projection, while posterior projection aneurysms located behind the ACoA were not evaluated in this plane due to being hidden. In the coronal plane, aneurysm projections on the reference lines were assessed in linear orientation as superior, inferior, medial, lateral, midline, and anterior. Aneurysm projections between the reference lines were evaluated in quadrant orientation and divided into superomedial, superolateral, inferomedial, and inferolateral quadrants.

In the axial plane, the reference lines were established using a parallel line passing through the ACoA and A1/Heubner’s as the *X* coordinate and a perpendicular line parallel to the A2s passing through the ACoA as the *Y* coordinate. The *Z* coordinate, which passes perpendicular to the *X* and *Y* coordinates from the ACoA and remains hidden, was used solely for the evaluation of superior projections. Aneurysms located above the ACoA were classified as superior projection, while inferior projection aneurysms located below the ACoA were not assessed in this plane due to being hidden. In this plane, the *X* coordinate was employed for anterior–posterior aneurysms (those anterior to the *X* coordinate were categorized as anterior), and the *Y* coordinate was used for medial–lateral projection aneurysms (those above the *Y* coordinate were classified as lateral, and those below were medial according to the midline, A1 dominance, and side of approach). In the axial plane, aneurysm projections with linear orientation on the reference lines were evaluated as anterior, posterior, lateral, medial, midline, and superior. Aneurysm projections between the reference lines were assessed in quadrant orientation and divided into anteromedial, anterolateral, posteromedial, and posterolateral quadrants (Fig. 1).

**Fig. 2:**
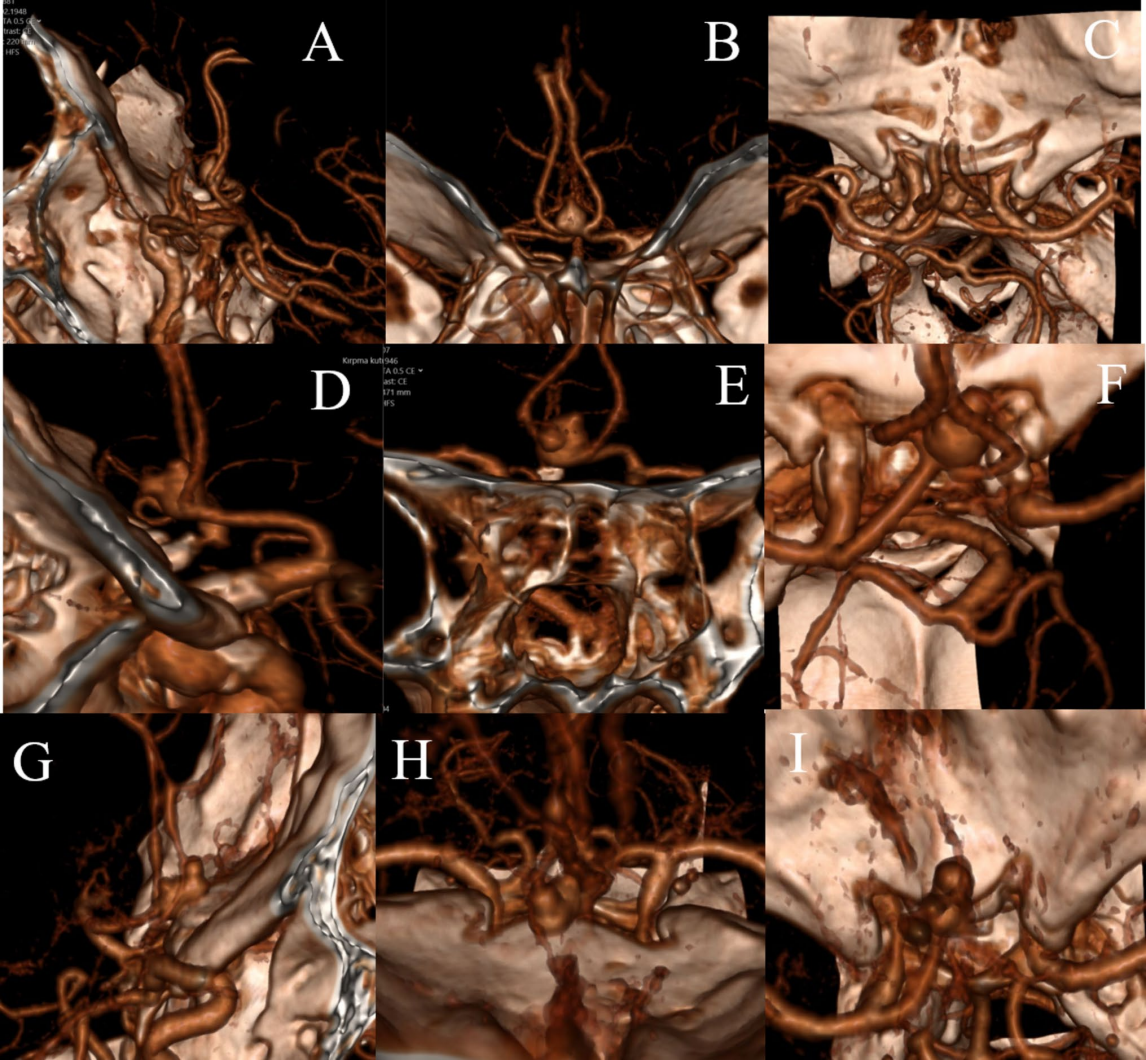
**A** ACoA aneurysm: true superior midline projection sagittal view. **B** Coronary view of true superior midline projection. **C** Axial view of true superior midline projection. **D** 3D CTA image of ACoA aneurysm with anterosuperior medial projection in sagittal view. **E** Coronal view of ACoA aneurysm with anterosuperior medial projection. **F** Axial view of ACoA aneurysm with anterosuperior medial projection. **G** Sagittal view of complex ACoA aneurysm. **H** Coronal view of complex ACoA aneurysm. **I** Axial view of complex ACoA aneurysm

Evaluation of aneurysm projection included these three planes and two orientations; those in linear orientation were performed in two dimensions and those in quadrant in three dimensions (superior midline, anterosuperior medial projection, etc.) (Fig. 2A–F).

If the aneurysm projection contained more than one quadrant or more than two linear orientations, it was considered a complex projection (Fig. 2G–I).

As a result, a 3D grading system comprising a total of 25 projections, including nine main projections with complex projections in four quadrants and four linear orientations in the sagittal plane, was created (Fig. 1D–F, Table 2).

**Table 2:** Our proposed novel projection classification system. Reference lines, advantages, and disadvantages of reference lines. Aneurysm projections (number of patients): linear and quadrant positions

Projections	Sagittal plane	Coronal plane	Axial plane
Linear	Superior Inferior Anterior Posterior	Superior Inferior Medial Lateral Median **Anterior**	Anterior Posterior Lateral Medial Median **Superior**
Quadrant	Anterosuperior Posterosuperior Anteroinferior Posteroinferior	Superomedial Superolateral Inferomedial Inferolateral	Anteromedial Anterolateral Posteromedial Posterolateral
	Complex		
	Referans lines		
*X*	Line parallel to the chiasm and ACoA perforans Those above are superior, and those below are inferior	Distal A1-proximal A2 junction, ACoA and the line and chiasm running perpendicular to the A2s in this plan and parallel to the ACoA perforans known to arise parallel to ACoA Those above are superior, and those below are inferior	Parallel line passing through ACoA and A1s Those in front are anterior, and the ones behind it are posterior
*Y*	Line from ACoA parallel to A2 proximal segments and perpendicular to the lamina terminalis Those in front are anterior, and the ones behind it are posterior	Perpendicular line passing through the ACoA in the midline and parallel to the A2s Those on the line, according to the midline, A1 dominance and approach side lateral ones lateral, medial ones media	Perpendicular line parallel to A2s passing through ACoA Those on the line, according to the midline, A1 dominance and approach side lateral ones lateral, medial ones media
*Z*	Med-lat (hidden)	Ant-post (hidden)	Sup-inf (hidden)
Advantages	Evaluates superior-inferior and anterior posterior projection	Allows assessment of superior-inferior and medial–lateral projections Allowing for anterior projection assessment	It provides an advantage in evaluating the aneurysm projection in interhemispheric interventions The proximal A1, proximal A2 outlets, and the aneurysm relationship are better seen in the axial plane as superposition may occur laterally It mainly provides anterior–posterior and medial lateral projection evaluation It also performs superior projection assessment A2 fork evaluation can be performed more accurately when the axial plane is added to the sagittal plane
Disadvantages	It does not allow medial–lateral-midline projection evaluation Insufficient in aneurysms with superior projection due to vascular superposition	The posterior projection is hidden in this plan (conventional AP imaging methods should not be used)	It does not show the inferior projection

Anterior in the coronal plane and superior in the axial plane are hidden as reference lines and can be visualized angiographically

In the presence of vertical A2 trace orientation, the anterior–posterior projection was replaced with the medial–lateral projection and the medial–lateral projection with the anterior–posterior projection owing to the 90-degree shift of the classical orientation. The anterior–posterior assessment was performed according to A1 dominance and the side of approach.

## Results

Of the 96 patients, 56 were men and 40 were women. Ninety-six cases were included, comprising 80 ruptured and 16 unruptured ACoA aneurysms.

The largest projections were posterosuperior (57.87), anteroinferior (57.85), and anterosuperior (57.05), while the smallest projections were posteroinferior (45), complex (46), and inferior (47).

All patients underwent 3D examination: CTA alone in 68 cases, DSA alone in 9 cases, MRA alone in 3 cases, CTA and DSA together in 13 cases, and CTA and MRA together in 3 cases.

The maximum diameters of the aneurysms ranged from 1.5 to 27 mm, with 35 aneurysms falling within the 5–7 mm range (35/96; 36.4%). Aneurysms less than 3 mm accounted for 5.2% (5/96), those between 3 and 5 mm for 27% (26/96), those between 7 and 10 mm for 21.8% (21/96), and those over 10 mm for 9.3% (9/96). The mean maximal aneurysm diameter was 6.3 mm in ruptured aneurysms and 7.91 mm in unruptured aneurysms. Aneurysm diameters exhibited variation based on projections, with the largest groups being 5–7 mm in the anterior projection (9/17; 52.9%), 3–5 mm in the anteroinferior projection (8/14; 57.1%), and 7–10 mm in the anterosuperior projection (13/34; 38.2%).

According to our classification system, 66.6% (64) of the aneurysm projections were located in the quadrant position, while 33.3% (32) were in the linear position. In the linear position, the anterior projection predominated with 17 aneurysms, followed by 9 with a superior projection, 4 with a posterior projection, and 2 with an inferior projection. In the quadrant position, the anterosuperior projection was the most prevalent, with 34 cases, followed by 14 with an anteroinferior projection, 8 with a posterosuperior projection, 2 with a posteroinferior projection, and 6 cases displaying a complex projection. In our study, overall, the distribution of aneurysm projections was as follows: 35.4% were anterosuperior, 17.7% were anterior, 14.58% were anteroinferior, 9.37% were superior, 8.3% were posterosuperior, 6.25% were complex, 4.16% were posterior, 2.08% were posteroinferior, and 2.08% were inferior.

Regarding the orientation of the aneurysm projections, 58.33% were found to be medial, 33.33% were midline, 5.2% were laterally oriented, and 3.25% were bilobulated.

The numbers and percentages of patients according to the aneurysm projection groups that were evaluated according to our own classification system are given in Table 3.

**Table 3:** Aneurysm projections (number of patients): linear and quadrant positions

Total (*n* = 96)	Projections	Number of case	%	Total number of projections %
Projection/linear position (*n* = 32)	Anterior	17	53	17.7
	Superior	9	28	9.37
	Posterior	4	12.5	4.16
	Inferior	2	6.25	2.08
Projection/quadrant position (*n* = 64)	Anterosuperior	34	53	35.4
	Anteroinferior	14	21.87	14.58
	Posterosuperior	8	12.5	8.33
	Posteroinferior	2	3.12	2.08
	Complex	6	9.37	6.25

Seventy-six patients (79%) presented with only ACoA aneurysms, while 20 patients (21%) had multiple aneurysms. Among the 80 patients in the ruptured group, 15 (18.75%) had multiple aneurysms, compared to 5 (31.25%) in the 16 patients in the unruptured group.

In ruptured aneurysms, 28 (35%) had an anterosuperior projection, 13 (16.25%) had an anteroinferior projection, and another 13 (16.25%) had an anterior projection. Among unruptured aneurysms, 6 (37.5%) had an anterosuperior projection, 4 (25%) had an anterior projection, and 2 (12.5%) had a posterosuperior projection.

Analyzing rupture rates based on aneurysm projections, the highest rupture rate was 92.85% (13/14) in the anteroinferior projection, followed by 82.35% (28/34) in the anterosuperior projection, and 76.47% (13/17) in the anterior projection.

In our study, A1 diameter asymmetry was identified in 78 cases (81.25%), with 41 cases (52.56%) demonstrating right A1 dominance and 37 cases (47.44%) exhibiting left A1 dominance. Symmetrical A1 diameters were observed in eighteen cases (18.75%). Among patients with right A1 dominance, 21 cases (51.22%) presented with marked left A1 hypoplasia, and 8 cases (19.51%) showed left A1 aplasia. Conversely, among those with left A1 dominance, 14 cases (37.84%) had marked right A1 hypoplasia, and 18 cases (48.65%) displayed right A1 aplasia.

Aneurysm projections were categorized as follows: 56 cases (58.33%) were medial, 32 cases (33.33%) were midline, 5 cases (5.2%) were laterally oriented, and 3 cases (3.25%) were bilobulated. Symmetrical A1 diameters were found in 44.44% of cases with midline aneurysm projection, 50% with medial orientation, and 1 case with lateral orientation. Among patients with A1 asymmetry, 60.25% had medial, 30.76% had midline, 5.12% had lateral aneurysm projections, and 3 patients presented with bilobulated aneurysms.

Regarding A2 orientation, 57 cases (59.37%) exhibited classical orientation, 24 cases (25%) showed oblique orientation, and 15 cases (15.62%) displayed vertical A2 orientation. Proximal A2 outlets were symmetrical in 76% of patients (73 cases), while 24% (23 cases) exhibited asymmetrical (open/closed) A2 forks.

While 70 cases (72.9%) underwent intervention, 26 (27.1%) cases were not treated for various reasons, including 17 with ruptured aneurysms and 9 with unruptured ones. Microsurgical clipping was performed in 61 out of the 70 cases (87.14%), and endovascular treatment was administered in the remaining 9 cases (12.86%). The distributions of projections for aneurysms treated endovascularly were as follows: 3 anterosuperior-medial, anterosuperior-midline, anteroinferior-medial, anterior-medial, anterior-lateral, posterosuperior-midline, and complex aneurysm projections.

When intervention methods were assessed based on aneurysm projections, clipping was utilized in 9 out of 11 anterior, 9 out of 10 anteroinferior, 24 out of 28 anterosuperior aneurysms, 3 out of 4 posterosuperior, and 4 out of 5 complex aneurysms, and all superior (6 cases), posterior (4 cases), and posteroinferior (2 cases) were surgically treated. Endovascular treatment is applied in the remaining 9 cases. For various reasons, 26 patients were not treated. The relationship between aneurysm projections and intervention methods according to our own classification system is given in Table 4.

**Table 4:** The distribution of patients was analyzed based on intervention status

	Intervention		
	Clipping	Endovascular	No intervention
Anterior (%)	9 (52.94)	2 (11.76)	6 (35.29)
Superior (%)	6 (66.6)	-	3 (33.3)
Posterior (%)	4 (100)	-	-
Inferior (%)	-	-	2 (100)
Anterosuperior (%)	24 (70.59)	4 (11.76)	6 (17.65)
Anteroinferior (%)	9 (64.28)	1 (7.14)	4 (28.57)
Posterosuperior (%)	3 (37.5)	1 (12.5)	4 (50)
Posteroinferior (%)	2 (100)	-	-
Complex (%)	4 (66.66)	1 (16.66)	1 (16.66)
Total (*n* = 96)	61 (63.54)	9 (9.38	26 (27.08)

Out of a total of 61 surgical cases, fenestrated clips were used in 10 cases: 3 anterosuperior-medial, 2 complex, 1 superior-midline, 1 anterosuperior lateral, 2 posterior medial, and 1 posteroinferior-medial (straight and right-angled projection). Additionally, 35 straight clips, 15 curved clips, 5 right-angled clips, and 1 bayonet clip were used. Multiple clips were used in 7 cases: 2 anterosuperior-midline, 2 posteroinferior-medial, 1 anteromedial, 1 anterosuperior-midline, and 1 complex projection. The rate of fenestrated clip use was high in projections with superior and posterior components.

Complications were evaluated on the basis of ACoA-related parent and/or perforating artery infarcts. The distribution among surgically treated ruptured aneurysm patients included 12 caudate (anteroinferior, 2 anterosuperior medial, posteroinferior medial, posterior-medial, superior-midline, superior-anteroinferior, superior medial, anterosuperior midline, posterosuperior-midline, anterior-medial, and anteroinferior-medial), two caudate + hypothalamic (2 anteroinferior midline), three hypothalamic (anterosuperior-medial, anteroinferior-midline, and anterior-midline), one hypothalamic + caudate + parent artery (DACA) infarct (anterosuperior medial), one DACA (complex), one bilateral hypothalamic + caudate infarct (posteroinferior-medial), and one hypothalamic + DACA infarct (anterosuperior-midline). There were no complications related to the parent or perforating arteries in the endovascularly treated patients with ruptured aneurysms.

No complications related to ACoA parent and/or perforating artery infarcts were encountered in patients with unruptured aneurysms treated surgically or endovascularly (5 in the surgical group and 2 in the endovascular group).

## Discussion

There are challenges in ACoA aneurysm surgery owing to different factors. In our study, we developed a new ACoA aneurysm classification system that identifies safe and dangerous zones using current 3D technology and reference lines based on important neurovascular structures in the region.

### Advantages of 3D CTA and DSA

Thanks to the advances in 3D technology, aneurysm morphology and projections can now be evaluated more accurately. While aneurysm projection can be assessed in conventional DSA in coronal and sagittal planes, 3D CTA and DSA allow a 360-degree global, coronal, sagittal, and axial view. It also allows a detailed evaluation of the relationship between ACoA aneurysm and proximal A1 and A2 outlets and the assessment of aneurysm projection from a desired surgical point of view. Therefore, the greatest advantage of 3D technology is the ability to visualize aneurysms and vascular structures in different positions with the desired surgical head position and perspective, thus contributing to the determination of the most appropriate surgical approach preoperatively.

### The importance of reference lines

**Fig. 3:**
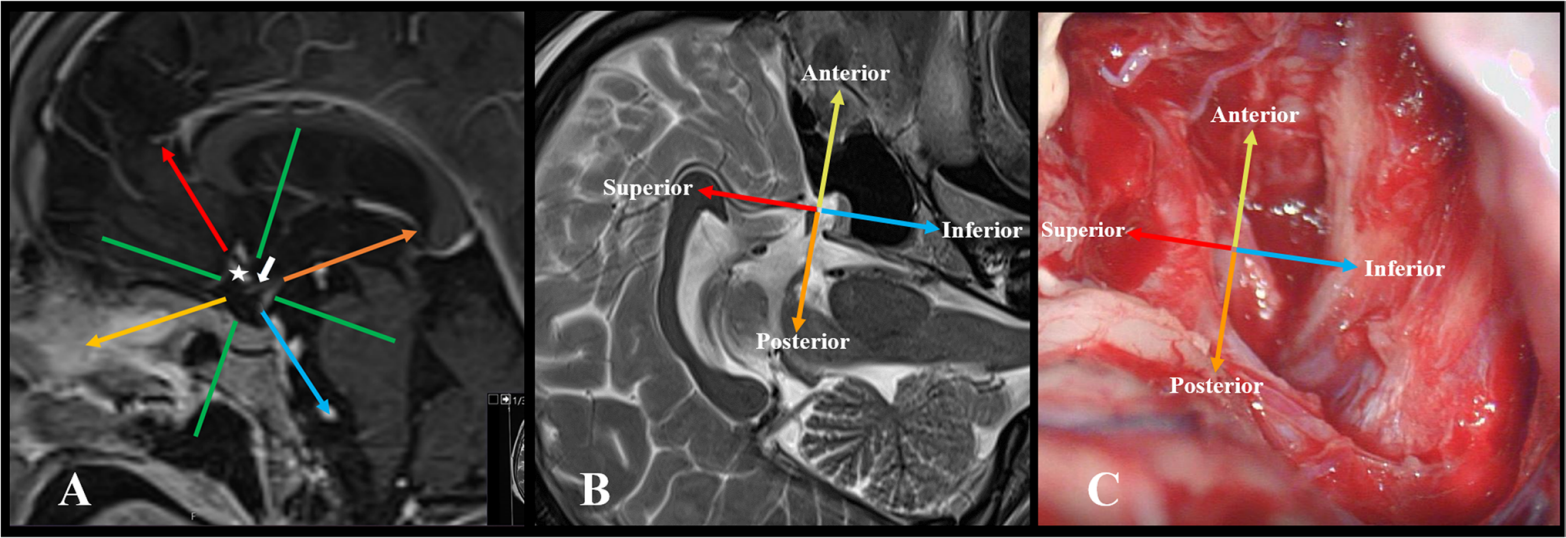
**A** Basal reference lines and reference lines based on neurovascular structures in sagittal plane MR (green line: basal line to the planum sphenoidale and the reference line perpendicular to it, yellow line: reference line passing through the chiasm, red line: reference line passing through the proximal A2s, orange line: reference line passing through the perforating arteries, blue line: reference line through the lamina terminalis, star: proximal A2s, and white arrow: optic chiasm). **B** Sagittal plane MR. Anterior: parallel to chiasma, inferior: lamina terminalis, and posterior: perforators. **C** Reference lines in the right pterional approach intraoperative view. Anterior: parallel to chiasma, inferior: lamina terminalis, and posterior: perforators

To date, there is no consensus on the classification of ACoA aneurysm projections. Various classifications have been proposed, and different reference lines have been used in these classifications. In some of these, the basal line and the line passing perpendicular to it from the ACoA were taken as the reference line, especially in the sagittal plane [3, 8, 9, 26–29], whereas in others, the line passing through the A2s and perpendicular to it was taken as the reference line [1, 5, 10, 11, 14, 18, 19, 21, 22, 25, 30–32, 36]. Therefore, it is necessary to take a common set of criteria as the basis for determining the reference lines. In our opinion, the determination of reference lines should be based on the vascular structures that constitute the ACoA complex and include A1/Heubner, A2, perforating arteries, and neural structures, such as optic chiasm and lamina terminalis. These will be viewed during surgery and will affect the surgical approach and technique. Indeed, bony structures, such as the planum sphenoidale or the anterior skull base, are not parallel to the chiasm tracing (Fig. 3). The fact that the aneurysm dome is considered anterior projection if it is parallel to the chiasm and inferior projection if it projects to the lamina terminalis indicates that neural structures as well as vascular anatomy may be reference points in aneurysm projection. This supports the projection evaluation based on neurovascular anatomy [1, 2, 5, 6, 9, 12, 13, 23, 32]. As it is directly related to clip placement, it is more appropriate for the vertical reference line to be parallel to the A2s in the sagittal plane. In this sense, aneurysms between A2s, which Hernesniemi [19] called “intertruncal” aneurysms, are those that best fit the definition of true superior projection. Again, since A2s are an important anatomical structure that is perpendicular to the chiasm, have the ease of separating the anterior and posterior quadrants, and affect the surgical technique, it seems more appropriate to call the aneurysms between A2s as “superior” in both surgical and anatomical classification. Moreover, when the basal line and the line perpendicular to it from ACoA are taken as the reference line as in many studies, and especially when quadrant evaluation is performed, an aneurysm that is located between or even behind A2s may be classified as an anterior projection aneurysm, which may lead to preoperative misjudgments in terms of surgical strategy and clipping technique [5, 14, 20, 26, 28].

The reason for using the line perpendicular to A2s as the horizontal reference line in the sagittal plane is that it runs parallel to the chiasm on the anterior side of ACoA. Furthermore, perforating arteries of ACoA, which must be preserved on the posterior side, are mostly perpendicular to A2s [32, 37–41].

### The importance of sagittal, coronal, and axial plans

To date, classifications regarding the projections of ACoAs have been made mostly in coronal and sagittal planes in conventional or 3D angiography [1, 3, 5, 8, 10, 11, 14, 17–22, 25, 29–33, 36], whereas axial projections have mostly been included in classifications for the interhemispheric approach [6, 8, 27, 28, 42].

In the evaluation of aneurysm projections, sagittal, coronal, and axial plans should be used concertedly. This is because each plan has advantages and disadvantages over the others (Table 2).

**Fig. 4:**
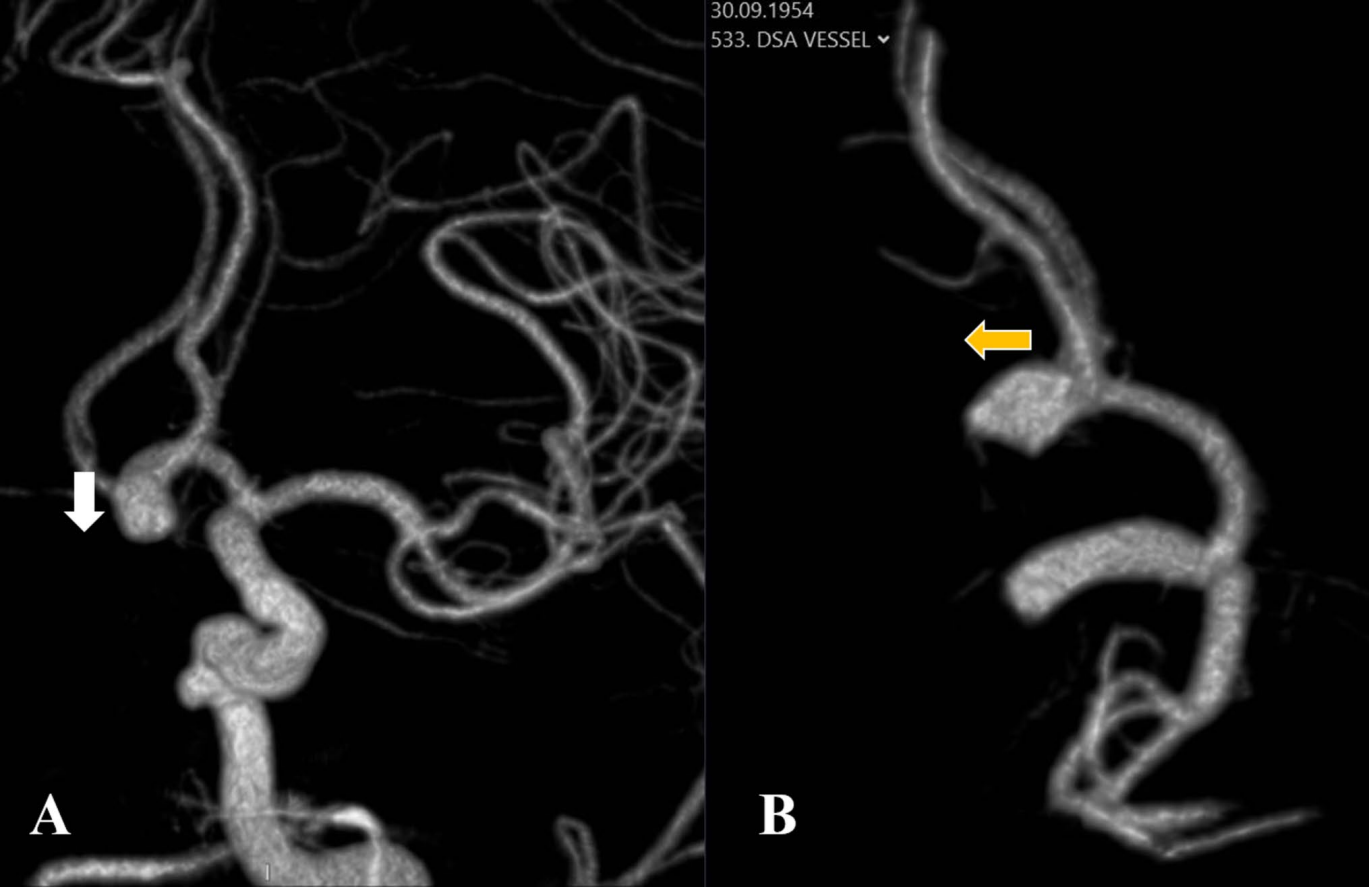
**A** AP image with inferior projection (white arrow) aneurysm appearance. **B** Aneurysm appearance that is understood to be anterior (yellow arrow) on lateral view

Without a projection evaluation that considers the reference lines, the projection may be misjudged and an anterior projection aneurysm may be mischaracterized as an inferior projection (Fig. 4).

In ACoA aneurysm projections in coronal and axial planes, the medial–lateral projection distinction varies according to A1 dominance and the side of approach. Therefore, the aneurysm dome is called median if it faces the dominant A1 or opposite the side of surgical approach, and it is called lateral if it faces the side of surgical approach.

### Advantages of quadrant orientation

#### Relationship between ACoA aneurysm projection and perforating arteries

**Fig. 5:**
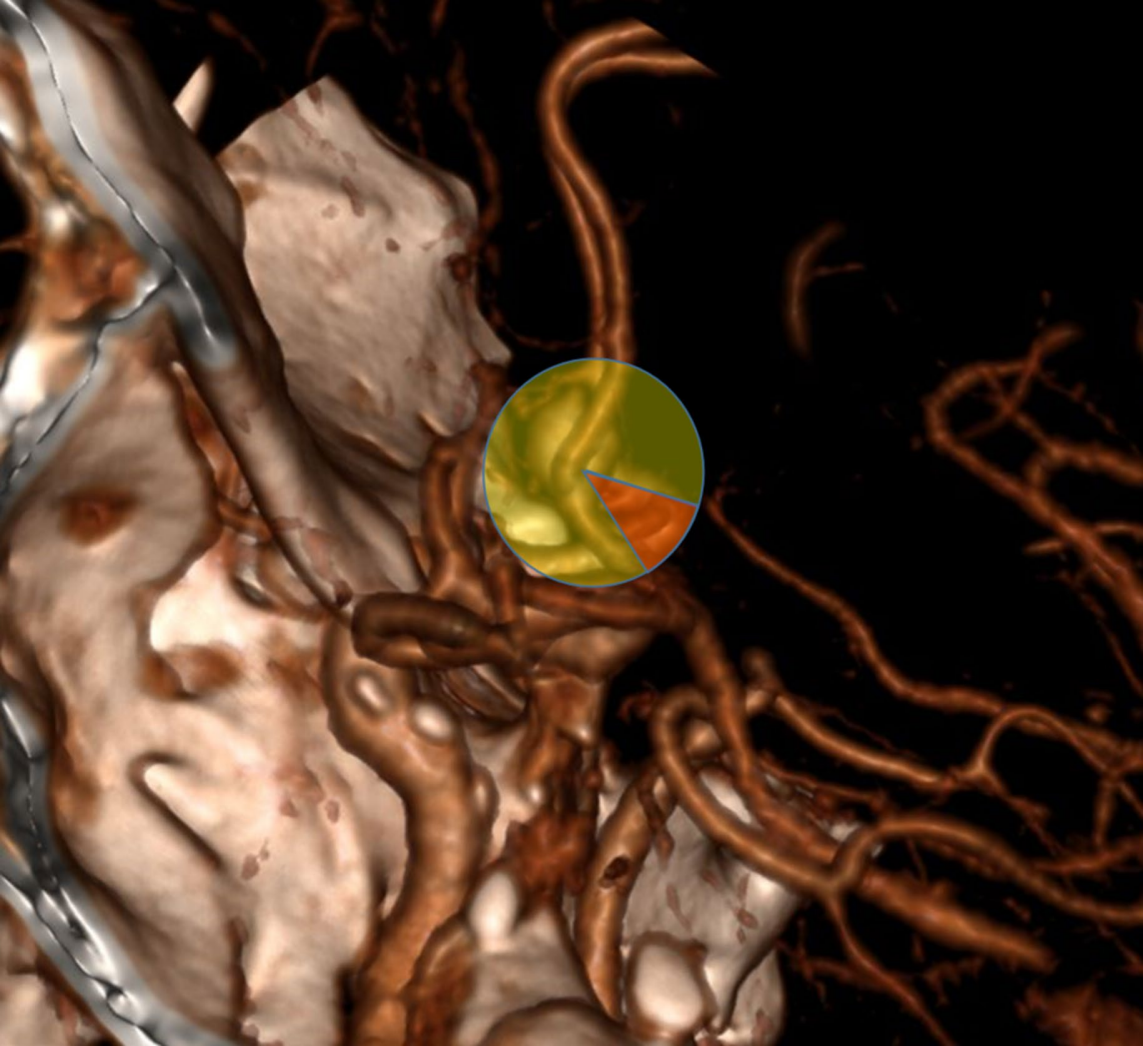
Safe and hazardous zones in sagittal plane. Orange area—safe zone, red area—hazardous zone

Often, ACoA projections are not in linear orientation only. The quadrant orientation also shows intermediate projection forms. Perforating arteries of ACoA originate from the posterior, superior, or inferior face of AcoA [13, 37, 39]. The subcallosal branch originates posterosuperiorly, whereas the hypothalamic branches and chiasmatic perforating arteries originate posteroinferiorly [40]. Marinkovic [38] reported that the subcallosal artery is present in 91% of the patients and that the subcallosal artery and small ACoA branches originate from the caudodorsal face of ACoA. Vincentelli [41] found the average angle between the perforating branches of ACoA and the A2 post-communicating segment to be 96 degrees (30–180) and between 90 and 120 degrees in 70% of the patients. Therefore, the posterior projection in linear orientation alone does not adequately describe the risk areas for perforations. It is more appropriate to identify safe and hazardous zones in the sagittal plane and quadrant orientation. The posteroinferior projection in the quadrant orientation better defines this hazardous zone. Therefore, quadrant orientation is preferable to linear orientation in defining the hazardous zone for perforations (Fig. 5).

### The advantage of linear orientation

If only quadrant orientation is used, there will be naming difficulties in determining which quadrant will include aneurysm projections in the linear orientation (e.g., intertruncal aneurysm between A2s). The main advantage of linear orientation is manifested in the superior projection where the aneurysm is located between A2s. In quadrant orientation, the clipping technique does not define the true superior aneurysm projection, which is particularly important in this projection, as well as the linear position.

Therefore, naming difficulties arise in the projections in the quadrants if evaluation is made in linear orientation and in the projections in the linear position if evaluation is made in quadrant orientation. Hence, it is more appropriate to make a classification that uses both linear and quadrant orientations together.

Moreover, it should be kept in mind that aneurysms may also have a thrombotic component and may cause intraoperative aneurysm projection changes.

### ACoA aneurysm projection classification systems

Various classification systems for ACoA aneurysm projections have been described in the literature. Although some studies are based on surgical orientation [18, 21, 24, 25, 34], most classification projection evaluations are based on anatomical orientation [3, 8, 9, 11, 14, 19, 28, 31] (Table 1).

The greatest shortcoming of classifications is that they include only the sagittal plane [1, 3, 5, 10–12, 14, 17, 19–22, 24, 25, 29–31, 33, 35, 36, 43]. The side of the projection was evaluated in unequal quadrant orientations in some studies [5, 20, 24], in equal quadrant orientations in some studies [3, 31, 33], in both linear and quadrant orientations in some studies [6, 13, 32], and only in the linear position in most studies [1, 2, 8, 9, 11, 17, 19, 21, 22, 24, 27–29, 35]. Ivan [8], on the contrary, emphasized a categorization based on the dimensions of both quadrants and octants.

In terms of dimension, AcoA aneurysms were classified in one dimension as anterior and posterior by Nathal [42], Proust [12], Gonzalez [35], Cai [33], and Shao [29] and as superior and inferior by Solomon [25] and Hyun [20]. Two-dimensional classifications were made as anterior, posterior, superior, and inferior [1, 3, 5, 10, 11, 14, 17, 19, 22, 25, 30, 31, 43]. 3D classifications include anterior, posterior, superior, and inferior, as well as medial and lateral projections [6, 8, 9, 18, 27, 28, 32].

There is also no consensus in the literature on where the reference lines should cross, but two views have been gaining significance. In the first view, the reference lines run parallel to A2s from ACoA in the sagittal plane and perpendicular to it at the level of AcoA [1, 2, 5, 10, 11, 14, 17–22, 25, 30–32]. In the second view, the transverse line in the sagittal plane runs parallel to the anterior skull base or planum sphenoidale and another reference line is drawn perpendicular to it at the level of the ACoA [3, 8, 9, 27–29, 33]. Again, there are two opinions about the reference line taken in the coronal plane. According to the first opinion, it is perpendicular to the midline from the ACoA [8, 32], and according to the other opinion, it is perpendicular to the A1-A2 junction [9, 28].

There are only two classifications in the literature that include three planes, three dimensions, and two orientations. In Batjer’s [32] classifications, linear and quadrant orientations are used, but the reference line is based on lines parallel and perpendicular to ACoA. In Ivan’s [8] classification, both parallel and perpendicular lines to the planum sphenoidale were taken as reference lines, quadrants and octanes were used as orientation rather than linear orientation, and 45-degree rotation was applied in the naming of quadrants. Additionally, neither classification includes the axial plan.

### ACoA aneurysm projection rates

The most common projections reported in the literature are superior and anterior projections [2, 5, 14, 20, 21, 42, 43]. The most frequently reported projections in the literature are superior and anterior. Yasargil [21] presented the following distribution based on his surgical classification: anterior projection (12.8%), superior projection (22.7%), posterior projection (34.4%), inferior projection (14.1%), and complex projection (16%). This surgical classification deviated 90 degrees from the anatomical classification. Similarly, Barlas et al. ^34^ found the posterior projection to be the most common (39%) in their anatomical classification, followed by the superior projection (22%). When aligned with the anatomical classification, superior aneurysms were the most prevalent.

Nathal [42] reported that superior aneurysms accounted for 58.9% and anterior aneurysms for 32.8%. Bohnstedt [1] also found the superior projection to be the most frequent (37.0%). In Suzuki’s [14] quadrant evaluation, the superior projection constituted 67.9%, while the inferior projection accounted for 32.1%.

According to Matsukawa [28], the anterior orientation is the most common. Moon [43] likewise reported anterior projection as the most prevalent, followed by superior, inferior, and posterior projections. In the quadrant position, Diraz [5] and Hyun [20] identified the anterior projection as the most common. Hyun [20] reported the distribution as follows: anterior projection (50.5%), inferior projection (24.2%), superior projection (19.2%), and posterior projection (6.1%). In the sagittal plane, Samson Batjer [32] identified the most common aneurysm as inferior or anterior.

According to our own classification system, two-thirds of the aneurysm projections were in quadrant orientation and one-third in linear orientation. Anterior projection in linear position (53%) and anterosuperior projection in quadrant position (53%) were the most common projections. When linear and quadrant orientations were evaluated together, the most common projection was anterosuperior (35.4%), followed by anterior (17.7%) and anteroinferior (14.58%) projections. Superior was seen in 9.37%, posterosuperior in 8.33%, complex in 6.25%, and posterior in 4.16%. The least common projections were inferior and posteroinferior projections with 2.08% **(**Table 3**)**.

Our proposed classification utilizes neurovascular structures that influence the surgical approach in the region as reference lines and includes three planes, three dimensions, and two orientations. Therefore, our aim is to create a classification that is understandable, surgically useful, and reliable, encompassing both safe and hazardous regions. Reference lines are shown in Fig. 1A–C.

### The importance of ACoA aneurysm projections

#### Relation to risk of rupture

Various studies have shown the correlation between morphological changes and rupture risk [28, 35, 43, 45]. Aneurysms with anterior projection [38, 43] and A1 asymmetry [35] indicate a higher rupture risk. Choi [27] found that superior projections are more prone to rupture, while superior projections have been associated with rebleeding [43]. Posterior aneurysms are larger, bleed less, and require more intraoperative angiography [34].

In our study, 80 cases involved ruptured aneurysms, with the majority presenting as anterosuperior (35%), anterior (16.25%), and anteroinferior (16.25%) projections. While the first two ranks remained consistent with the unruptured aneurysm projections, the posterosuperior projection took the third place in unruptured cases. However, due to the parallel distribution of rupture rates with the frequency of projections and the limited number of unruptured cases, we were unable to draw definitive conclusions about the relationship between specific projections and a higher risk of rupture.

#### Relation to surgical strategy

Different projections pose unique challenges. Inferior projections limit proximal control due to obscuration of the contralateral A1 segment, while anterior projections hinder dissection of the distal aneurysm neck and contralateral A1–A2 junction. Superior projections may obscure the contralateral A2 segment, and posterior projections conceal the ACoA perforating arteries [3, 18, 37]. Surgical strategies such as fenestrated clips or creative clip configurations may be required for superior and posterior projections [3, 34].

Anterior and inferior projections often involve adherence to the optic nerve or chiasm, posing risks during frontal lobe exclusion. The anterosuperior projection often shows adhesion to A2 segments, while superoposterior projections are concealed behind parent arteries and the gyrus rectus, which reduces the risk of hypothalamic perforans [1–3, 37, 39, 44]. In the posterior projection, contralateral A1 and Heubner are at risk, and inferior aneurysms are adherent to the optic apparatus. Resection of the gyrus rectus may be required for superior and posterior aneurysms [34]. Proust [12] highlighted the high risk of injury to perforating arteries, parent artery, and ACoA in posterior projections.

Clip selection depends on aneurysm size, projection, and involvement of perforating arteries [37]. Fenestrated clips are more frequently used in superior and posterior projections (50.4% and 57.1% on A2s, and 16.5% on A1 in the inferior projection) [42]. In our case series, fenestrated clips were used in 10 cases, and the rate of use of fenestrated clips is high in projections involving superior and posterior components.

We believe that the surgical difficulties and risks related to the projections in the existing classification systems in the literature are valid. In addition, we think that both posterior projections in the linear plane and posteroinferior projections in the quadrant plane are more challenging in terms of perforating damage according to our proposed classification system. As suggested in the literature and as we have seen in our case series, we believe that the use of fenestrated clips may be necessary in projections involving superior, posterior, and inferior components according to our proposed classification system. However, this information should be supported by further prospective large series.

#### Relation to the type of intervention

Aneurysm projection plays a vital role in determining treatment options for ACoA aneurysms. Endovascular approaches are recommended for posterior projections due to the risks associated with clipping, including potential injury to the posterior perforating artery, parent artery, and ACoA, as well as the need for gyrus rectus resection [27–30]. Coil placement is commonly favored for posterior projections [15, 31]. Aneurysm projection can also impact the completeness of occlusion in embolization procedures. Several studies have shown that superior and posterior dome orientations are associated with incomplete occlusion [15, 17, 19, 24, 33].

In our case series, 72.9% underwent intervention and 27.1% of cases were not treated for various reasons. Microsurgical clipping was performed in 87.14% of the patients who underwent intervention, while endovascular treatment was performed in the remaining 12.86% of patients. Different projections were observed in 9 patients who underwent endovascular treatment (3 anterosuperior-medial, anterosuperior-midline, anteroinferior-medial, anterior-medial, complex, anterior-lateral, and posterosuperior-midline). Therefore, when the treatment choice was evaluated, it was understood that the planning was not done according to the aneurysm projection.

In our treatment approach for cases with AcoA aneurysms, the consideration of aneurysm projection was tempered by several factors. Priority was given to the patient’s WFNS score, the urgency or ultra-urgency of intervention, presence of aneurysm rupture, surgical experience of the clinic, the feasibility of endovascular procedures, and the preferences of the patient and their relatives. As a result, although aneurysm projection was taken into account, its influence on the treatment approach was often secondary. Consequently, establishing a direct relationship between aneurysm projection and the applied treatment approach proved challenging.

According to our proposed classification system, in light of the literature, it is recommended that endovascular treatment should be prioritized in the treatment of not only aneurysms with posterior projection but also aneurysms with posteroinferior projection within the region we define as the hazardous zone when projection-based treatment is planned. However, large case series are needed to support this, using the classification system we propose.

#### Relation to the complications

Huang [36] emphasized the usefulness of ACoA projection in predicting complications. Chen [3] highlighted that aneurysm orientation not only impacts surgical decisions but also influences the surgical approach, exposure difficulty, clipping efficacy, and overall surgical complexity. Prognosis-wise, posterior, and superior aneurysms have worse outcomes, while anteroinferior aneurysms have better grades and prognosis [1, 26].

In our case series, complications were evaluated on the basis of ACoA-related infarcts in the parent and/or perforator arteries. In patients with surgically treated ruptured aneurysms, there was no association between vascular complications and aneurysm projection. In the endovascularly treated ruptured aneurysm patients, there were no complications related to parent or perforating arteries.

No complications related to ACoA parent and/or perforating artery infarcts were encountered in patients with unruptured aneurysms treated surgically or endovascularly (5 in the surgical group and 2 in the endovascular group). The absence of vascular complications in unruptured aneurysms, whether surgical or endovascular, suggests that other pathologies related to rupture (such as primary injury and/or vasospasm) are more effective than projection in the development of complications. In addition to aneurysm projection, the effect of factors such as surgeon experience, surgical technique, WFNS score, side and method of surgical approach, and vasospasm should be considered in the development of complications in ruptured aneurysms.

### Limitations of the study

The main goal of this study was to develop a classification for ACoA aneurysms using 3D images provided by modern imaging modalities to overcome the shortcomings of previous classifications. The radiological and clinical data used in this study were retrospective and were used to develop this classification. Therefore, it is recommended to assess the clinical implications of the study with a larger case series created using the projection classification system proposed in this study.

## Conclusion

The projection of ACoA aneurysms is a crucial factor in surgical decision-making, surgical approach, techniques employed, potential complications, and, ultimately, patient outcomes. Our study holds significant value as it introduces a novel preoperative projection classification utilizing 3D angiography, which is widely employed in clinical practice. This classification system incorporates both linear and quadrant orientations, providing a comprehensive evaluation of aneurysm projections.

By leveraging 3D technology, our proposed classification aims to enhance surgical safety by considering intricate neurovascular structures. It enables a thorough assessment of safe and hazardous zones across three planes, three dimensions, and two orientations, empowering surgeons to gain a detailed understanding of ACoA aneurysms. This knowledge can facilitate precise surgical planning and execution, potentially leading to improved patient outcomes.

## Data Availability

The data and materials supporting the findings of this study are available from Trakya University Hospital upon reasonable request. Researchers interested in accessing the data and materials used in this study should contact morakdogen@gmail.com for further information.

## References

[1] Agrawal A, Kato Y, Chen L, Karagiozov K, Yoneda M, Imizu S, Sano H, Kanno T (2008) Anterior communicating artery aneurysms: an overview. Min-Minim Invasive Neurosurg 51(3):131–135. 10.1055/s-2008-1073169 10.1055/s-2008-1073169 18521782

[2] Bohnstedt BN, Conger AR, Edwards J, Ziemba-Davis M, Edwards G, Brom J, Shah K, Cohen-Gadol AA (2019) Anterior communicating artery complex aneurysms: anatomic characteristics as predictors of surgical outcome in 300 cases. World Neurosurg 122:e896–e906. 10.1016/j.wneu.2018.10.172 30404067 10.1016/j.wneu.2018.10.172

[3] Chen J, Li M, Zhu X, Chen Y, Zhang C, Shi W, Chen Q, Wang Y (2020) Anterior communicating artery aneurysms: anatomical considerations and microsurgical strategies. Front Neurol 11:1020. 10.3389/fneur.2020.01020 33013671 10.3389/fneur.2020.01020 PMC7509403

[4] Debono B, Proust F, Langlois O, Clavier E, Douvrin F, Derrey S, Freger P (2004) Ruptured anterior communicating artery aneurysm. Therapeutic options in 119 consecutive cases. Neuro-chirurgie 50(1):21–32. 10.1016/s0028-3770(04)98302-6 15097917 10.1016/s0028-3770(04)98302-6

[5] Diraz A, Kobayashi S, Toriyama T, Ohsawa M, Hokama M, Kitazama K (1993) Surgical approaches to the anterior communicating artery aneurysm and their results. Neurol Res 15(4):273–280. 10.1080/01616412.1993.11740148 8105408 10.1080/01616412.1993.11740148

[6] Inagawa T (1999) Dissection from fundus to neck for ruptured anterior and middle cerebral artery aneurysms at the acute surgery. Acta Neurochir 141:563–570. 10.1007/s007010050344 10929720 10.1007/s007010050344

[7] Ito H, Onodera H, Wakui D, Uchida M, Sase T, Morishima H, Oshio K, Tanaka Y (2016) Impact of aneurysmal neck position in endovascular therapy for anterior communicating artery aneurysms. Neurol Med Chir 56(1):21–26. 10.2176/nmc.oa.2015-0201 10.2176/nmc.oa.2015-0201 PMC4728145 26458847

[8] Ivan ME, Safaee MM, Martirosyan NL, Rodríguez-Hernández A, Sullinger B, Kuruppu P, Habdank-Kolaczkowski J, Lawton MT (2019) Anatomical triangles defining routes to anterior communicating artery aneurysms: the junctional and precommunicating triangles and the role of dome projection. J Neurosurg 132(5):1517–1528. 10.3171/2018.12.JNS183264 30952121 10.3171/2018.12.JNS183264

[9] Kasinathan S, Yamada Y, Cheikh A, Teranishi T, Kawase T, Kato Y (2019) Prognostic factors influencing outcome in unruptured anterior communicating artery aneurysm after microsurgical clipping. Asian J Neurosurg 14(1):28–34. 10.4103/ajns.AJNS_198_18 30937004 10.4103/ajns.AJNS_198_18 PMC6417356

[10] Kato Y, Nouri M, Shu G (2019) Surgery of anterior communicating artery aneurysms. In: July J, Wahjoepramono EJ (ed) Neurovascular Surgery: Surgical Approaches for Neurovascular Diseases. Springer, Singapore, pp 117–124. 10.1007/978-981-10-8950-3

[11] Lawton MT (2011) Seven aneurysms: tenets and techniques for clipping. Thieme. 68(6):1774. 10.1227/NEU.0b013e31821819b9 10.1227/NEU.0b013e31821819b9 21389888

[12] Proust F, Debono B, Hannequin D et al (2003) Treatment of anterior communicating artery aneurysms: complementary aspects of microsurgical and endovascular procedures. J Neurosurg 99(1):3–14. 10.3171/jns.2003.99.1.0003 12854737 10.3171/jns.2003.99.1.0003

[13] Sekhar LN, Natarajan SK, Britz GW, Ghodke B (2007) Microsurgical management of anterior communicating artery aneurysms. Oper Neurosurg 61(5):273–292. 10.1227/01.neu.0000303980.96504.d9 10.1227/01.neu.0000303980.96504.d9 18091242

[14] Suzuki M, Fujisawa H, Ishihara H, Yoneda H, Kato S, Ogawa A (2008) Side selection of pterional approach for anterior communicating artery aneurysms–surgical anatomy and strategy. Acta Neurochir 150:31–39. 10.1007/s00701-007-1466-9 18058058 10.1007/s00701-007-1466-9

[15] Tarulli E, Fox A (2010) Potent risk factor for aneurysm formation: termination aneurysms of the anterior communicating artery and detection of A1 vessel asymmetry by flow dilution. Am J Neuroradiol 31(7):1186–1191. 10.3174/ajnr.A2065 20360345 10.3174/ajnr.A2065 PMC7965473

[16] Uemura A, Kamo M, Matsukawa H (2012) Angiographic outcome after endovascular therapy for anterior communicating artery aneurysms: correlation with vascular morphological features. Jpn J Radiol 30:624–627. 10.1007/s11604-012-0099-y 22760947 10.1007/s11604-012-0099-y

[17] Bhattarai R, Liang C-F, Chen C, Wang H, Huang T-C, Guo Y (2020) Factors determining the side of approach for clipping ruptured anterior communicating artery aneurysm via supraorbital eyebrow keyhole approach. Chin J Traumatol 23(1):20–24. 10.1016/j.cjtee.2019.12.002 32081450 10.1016/j.cjtee.2019.12.002 PMC7049606

[18] Chen L, Agrawal A, Kato Y, Karagiozov KL, Kumar MV, Sano H, Kanno T (2009) Role of aneurysm projection in “A2” fork orientation for determining the side of surgical approach. Acta Neurochir 151:925–933. 10.1007/s00701-009-0407-1 19499172 10.1007/s00701-009-0407-1

[19] Hernesniemi J, Dashti R, Lehecka M, Niemelä M, Rinne J, Lehto H, Ronkainen A, Koivisto T, Jääskeläinen JE (2008) Microneurosurgical management of anterior communicating artery aneurysms. Surg Neurol 70(1):8–28. 10.1016/j.surneu.2008.01.056 18452980 10.1016/j.surneu.2008.01.056

[20] Hyun SJ, Hong SC, Kim JS (2010) Side selection of the pterional approach for superiorly projecting anterior communicating artery aneurysms. J Clin Neurosci 17(5):592–596. 10.1016/j.jocn.2009.09.024 20223671 10.1016/j.jocn.2009.09.024

[21] Yașargil MG, (1984) Clinical considerations, surgery of the intracranial aneurysms and results. Microneurosurgery 2:33–123

[22] Petraglia AL, Srinivasan V, Moravan MJ, Coriddi M, Jahromi BS, Vates GE, Maurer PK (2011) Unilateral subfrontal approach to anterior communicating artery aneurysms: a review of 28 patients. Surg Neurol Int 2:124. 10.4103/2152-7806.85056 22059119 10.4103/2152-7806.85056 PMC3205488

[23] Riina HA, Lemole GM Jr, Spetzler RF (2002) Anterior communicating artery aneurysms. Neurosurg 51(4):993–996. 10.1227/01.NEU.0000027765.68754.6B 10.1097/00006123-200210000-00026 12234409

[24] Solomon RA (2001) Anterior communicating artery aneurysms. Neurosurg 48(1):119–123. 10.1097/00006123-200101000-00021 10.1097/00006123-200101000-00021 11152337

[25] Wang H, Chen C, Ye Z-P, Luo L, Li W-S, Guo Y (2015) On clipping of anterior communicating artery aneurysm via eyebrow-lateral keyhole approach. Int J Clin Exp Med 8(11):21114 26885043 PMC4723888

[26] Cai W, Hu C, Gong J, Lan Q (2018) Anterior communicating artery aneurysm morphology and the risk of rupture. World Neurosurg 109:119–126. 10.1016/j.wneu.2017.09.118 28958928 10.1016/j.wneu.2017.09.118

[27] Choi JH, Jo KI, Kim KH, Jeon P, Yeon JY, Kim JS, Hong SC (2016) Morphological risk factors for the rupture of anterior communicating artery aneurysms: the significance of fenestration. Neuroradiol 58:155–160 10.1007/s00234-015-1610-9 26511858

[28] Matsukawa H, Uemura A, Fujii M, Kamo M, Takahashi O, Sumiyoshi S (2013) Morphological and clinical risk factors for the rupture of anterior communicating artery aneurysms. J Neurosurg 118(5):978–983. 10.3171/2012.11.JNS121210 23240701 10.3171/2012.11.JNS121210

[29] Shao X, Wang H, Wang Y, Xu T, Huang Y, Wang J, Chen W, Yang Y, Zhao B (2016) The effect of anterior projection of aneurysm dome on the rupture of anterior communicating artery aneurysms compared with posterior projection. Clin Neurol Neurosurg 143:99–103. 10.1016/j.clineuro.2016.02.023 26914141 10.1016/j.clineuro.2016.02.023

[30] Birknes JK, Hwang S-K, Pandey AS, Cockroft K, Dyer AM, Benitez RP, Veznedaroglu E, Rosenwasser RH (2006) Feasibility and limitations of endovascular coil embolization of anterior communicating artery aneurysms: morphological considerations. Neurosurgery 59(1):43–52. 10.1227/01.neu.0000243282.60673.31 16823299 10.1227/01.NEU.0000219220.25721.B9

[31] Nossek E, Setton A, Karimi R, Dehdashti AR, Langer DJ, Chalif DJ (2016) Analysis of superiorly projecting anterior communicating artery aneurysms: anatomy, techniques, and outcome. A proposed classification system. Neurosurg Rev 39:225–235. 10.1007/s10143-015-0677-4 26631225 10.1007/s10143-015-0677-4

[32] Samson D, Batjer HH, White J, Trammell T, Eddleman CS (2011) Aneurysms of theAnterior Communicating Artery. In: Samson D, Batjer HH, White J, Trammell T, Eddleman CS (eds) Intracranial aneurysm surgery: basic principles and techniques. Thieme, New York, pp 88–109

[33] Cai W, Shi D, Gong J, Chen G, Qiao F, Dou X, Li H, Lu K, Yuan S, Sun C, Lan Q (2015) Are morphologic parameters actually correlated with the rupture status of anterior communicating artery aneurysms? World Neurosurg 84(5):1278–1283. 10.1016/j.wneu.2015.05.060 26054869 10.1016/j.wneu.2015.05.060

[34] Barlas O, Çoban O, Hepgül KT, Meltem C, Nail İ (1994) Fundus direction and vascular anomalies associated with anterior communicating artery aneurysms. Turk Neurosurg 4(2):67–72

[35] Gonzalez N, Sedrak M, Martin N, Vinuela F (2008) Impact of anatomic features in the endovascular embolization of 181 anterior communicating artery aneurysms. Stroke 39(10):2776–2782. 10.1161/STROKEAHA.107.505222 18617670 10.1161/STROKEAHA.107.505222

[36] Huang Judy GAV, Tamargo Rafael JHR (2011) Neurological Surgery, 4-volume set. In: Youmans JR, Winn HR (eds) Anterior Communicating Artery Aneurysms, 6th edn. Elsevier, New York, p 3841

[37] Kedia S, Daisy S, Mukherjee KK, Salunke P, Srinivasa R, Narain MS (2013) Microsurgical anatomy of the anterior cerebral artery in Indian cadavers. Neurol India 61(2):117. 10.4103/0028-3886.111113 23644309 10.4103/0028-3886.111113

[38] Marinković S, Milisavljević M, Marinković Z (1990) Branches of the anterior communicating artery: microsurgical anatomy. Acta Neurochir 106:78–85. 10.1007/BF01809337 2270791 10.1007/BF01809337

[39] Perlmutter D, Rhoton AL (1976) Microsurgical anatomy of the anterior cerebral-anterior communicating-recurrent artery complex. J Neurosurg 45(3):259–272. 10.3171/jns.1976.45.3.0259 948013 10.3171/jns.1976.45.3.0259

[40] Serizawa T, Saeki N, Yamaura A (1997) Microsurgical anatomy and clinical significance of the anterior communicating artery and its perforating branches. Neurosurgery 40(6):1211–1218. 10.1097/00006123-199706000-00019 9179894 10.1097/00006123-199706000-00019

[41] Vincentelli F, Lehman G, Caruso G, Grisoli F, Rabehanta P, Gouaze A (1991) Extracerebral course of the perforating branches of the anterior communicating artery: microsurgical anatomical study. Surg Neurol 35(2):98–104. 10.1016/0090-3019(91)90258-b 1990488 10.1016/0090-3019(91)90258-b

[42] Nathal E, Yasui N, Sampei T, Suzuki A (1992) Intraoperative anatomical studies in patients with aneurysms of the anterior communicating artery complex. J Neurosurg 76(4):629–634. 10.3171/jns.1992.76.4.0629 1545257 10.3171/jns.1992.76.4.0629

[43] Moon K, Levitt MR, Almefty RO, Nakaji P, Albuquerque FC, Zabramski JM, McDougall CG, Spetzler RF (2015) Treatment of ruptured anterior communicating artery aneurysms: equipoise in the endovascular era? Neurosurgery 77(4):566–571. 10.1227/NEU.0000000000000878 26308643 10.1227/NEU.0000000000000878

[44] Brzegowy P, Kucybała I, Krupa K, Łasocha B, Wilk A, Latacz P, Urbanik A, Popiela TJ (2019) Angiographic and clinical results of anterior communicating artery aneurysm endovascular treatment. Videosurgery Other Miniinvasive Tech 14(3):451–460. 10.5114/wiitm.2019.81406 10.5114/wiitm.2019.81406 PMC6748064 31534577

[45] Lin N, Ho A, Charoenvimolphan N, Frerichs KU, Day AL, Du R (2013) Analysis of morphological parameters to differentiate rupture status in anterior communicating artery aneurysms. PLoS One 8(11) 10.1371/journal.pone.0079635 PMC3827376 24236149

